# Risk for Surgical Team Hearing Loss With Vitrectomy

**DOI:** 10.1177/24741264231172564

**Published:** 2023-05-17

**Authors:** Sunil Ruparelia, Samantha Orr, Netan Choudhry, Robert W. Wong, Corey A. Smith, S. Mark Taylor, R. Rishi Gupta

**Affiliations:** 1 Dalhousie Medical School, Halifax, NS, Canada; 2 Vitreous Retina Macula Specialists of Toronto, Etobicoke, ON, Canada; 3 Octane Imaging Laboratory, Toronto, ON, Canada; 4 University of Toronto, Toronto, ON, Canada; 5 Ascension Seton Medical Center Austin, Austin, TX, USA

**Keywords:** vitrectomy, retina, hearing loss, occupational health

## Abstract

**Purpose:** To assess sound-level exposure during vitrectomy using 3 of the most common commercially available machines. **Methods:** This noninterventional cross-sectional study examined sound emission from the Constellation, Stellaris, and EVA vitrector systems. For each machine, a noise dosimeter was used to measure the sound-level exposure of the surgeon during 3 surgical cases in which vitrectomy was performed. Sound levels associated with progressively increasing cut rates and vacuum pressures were also measured. Finally, sound measurements were taken during the use of various additional functions of each machine, including diathermy, laser, and extrusion. Sound levels were compared with occupational health guidelines in Canada and the United States. **Results:** The maximum sound level recorded during vitrectomy surgery was 88.2 dBA. The mean sound level during vitrectomy surgical cases ranged from 58.5 to 66.8 dBA. A strong positive linear correlation was found between the cut rate and sound level (*r* = 0.88-0.98) and the vacuum pressure and sound level (*r* = 0.83-0.97). This relationship was consistent across the 3 vitrector systems (*P* < .001). **Conclusions:** Noise exposure during vitrectomy procedures was acceptable but may be sufficient for surgical team activity interference, as described by World Health Organization recommendations. A strong correlation was found between the cut rate and noise exposure. If cut rates continue to increase, attention should be given to ensure that the resulting noise exposure does not threaten the hearing of vitreoretinal surgeons and the operating room staff.

## Introduction

Vitrectomy is the most common surgical procedure performed by the vitreoretinal specialist. The vitreous cutter has evolved significantly since Robert Machemer’s first designs in the 1970s.^1^ The modern vitrector uses a pneumatic guillotine, which operates with an outer needle and an inner needle. This pneumatic-driven system cuts the vitreous while simultaneously aspirating severed strands. A separate infusion line is used to maintain intraocular pressure during the procedure.^2^

Cut rates have increased progressively over the past 2 decades. Whereas 1500 cuts per minute (cpm) was standard in the early 1990s, modern technology allows for upward of 10 000 cpm, with 20 000 cpm cutters beginning to enter practice.^3^ This increase in the cut rate may be associated with increasing sound levels produced by the vitrector and cutter apparatus. Furthermore, whether this level of noise risks hearing damage has thus far not been investigated to our knowledge.

Hearing loss is the fourth leading contributor to disability worldwide.^4^ A common etiology of sensorineural hearing loss is noise-induced hearing loss, which is prevalent in North America. A study found that approximately 28 million people in the US have impaired hearing, and around half of these cases were attributed to noise exposure.^5^ Levels of occupational noise exposure are of particular concern because exposure often occurs frequently and for long durations.^6^ Devastating, noise-induced hearing loss is often preventable. A better understanding of potential exposure settings may contribute to a reduction in occupational hearing loss.

Existing research has shown potentially damaging sound levels in other surgical settings.^7^ However, to our knowledge there are no studies in the existing literature that have evaluated the risk for hearing loss during any ophthalmology surgery. This study sought to determine whether vitrectomy surgery with a guillotine cutter produces sound levels significant enough to be associated with long-term hearing loss in surgical staff.

## Methods

This multicenter study was performed at the following 3 major centers in North America: Victoria General Hospital, Halifax, Nova Scotia, Canada; Burlington Laser Eye Centre, Toronto, Ontario, Canada; and Dell Medical Center, Austin, Texas, USA. The study received approval from the respective institutional ethics review boards and was conducted in accordance with the Declaration of Helsinki.

The Constellation (Alcon, Inc), Stellaris (Bausch + Lomb), and EVA (Dorc, USA) vitrector systems were used at the Halifax, Toronto, and Austin sites, respectively. The Extech SL400 noise dosimeter and calibrator (ITM) was used to record the sound-level exposure at each site. The same dosimeter was used at each site and was recalibrated in each operating room (OR) to ensure consistency among measurements.

**Figure 1.**
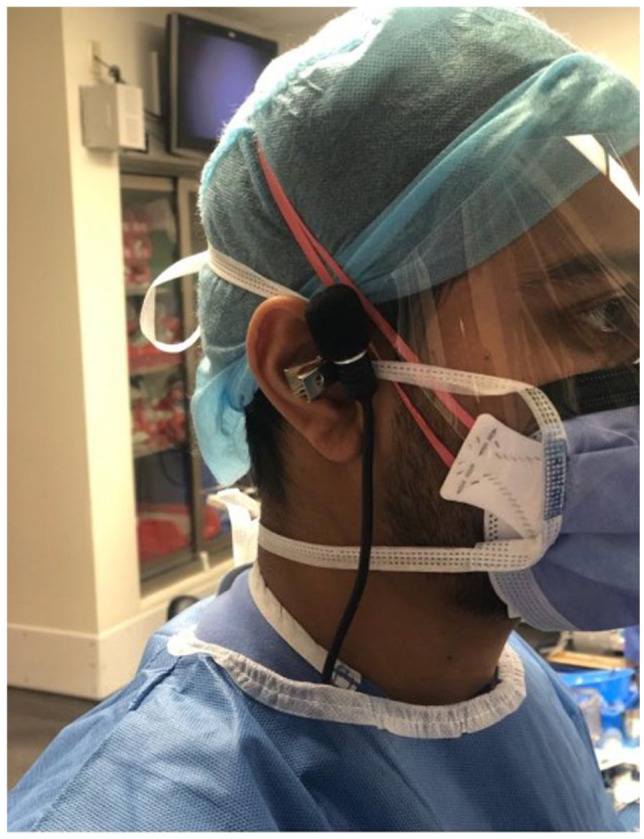
Microphone placement on surgeon’s mask for sound-level recordings during surgery, measured 2.5 cm (1 inch) from the ear.

For each vitrector system, sound levels from the cutter during 3 in vivo 25-gauge vitrectomies were recorded. Procedures included were vitrectomy for vitreous hemorrhage, epiretinal membrane peeling, retinal detachment, or macular hole repair. The cut rates used during these procedures were adjusted at the preference of the surgeon to be reflective of a routine case. The primary surgeon of the case was equipped with the noise dosimeter. The dosimeter was attached under the surgeons’ gown and the microphone attached to the surgeons’ mask, approximately 2.5 cm from the ear (Figure 1).

To accurately portray the human hearing response curve, sound levels were recorded by the dosimeter on an A-weighted (dBA) scale. The baseline OR sound level was measured and recorded before each surgical case. The distance from the surgeon to the vitrector was measured before each case and was consistent between sites.

To evaluate the relationship between the cut rate and the sound level, sound emitted from the vitrector systems with 23-gauge, 25-gauge, and 27-gauge cutters at progressively increasing cut rates was also measured and plotted. These measurements were taken with ambient noise minimized in the OR setting (without conversation, movement, or monitor alarms). At each cut rate, 10 sound level recordings taken with the cutter inside the eye were obtained and the average sound level was plotted. Sound produced by the vitrector during vacuum function at increasing vacuum pressures was similarly measured and recorded. Sound levels produced artificially from the vitrector machine itself were measured during various functions, including reflux, viscous fluid control (VFC) extract, VFC inject laser, diathermy, and voice naming procedure. Sound levels were compared with current Canadian Centre for Occupational Health and Safety (CCOHS) guidelines and US National Institute for Occupational Safety and Health (NIOSH) guidelines.^8,9^ Table 1 summarizes these guidelines.

**Table 1.** Guidelines for Noise Exposure Listed by the US National Institute for Occupational Safety and Health and the Canadian Centre for Occupational Health and Safety.^8,9^

Allowable Sound Level (dBA)	Maximum Permitted Daily Duration (Hours)
85	8.00
88	4.00
91	2.00
94	1.00
97	0.50
100	0.25

Statistical analysis was performed using SPSS Statistics for Macintosh (version 27.0, IBM Corp) and Prism software (version 9.1.2, GraphPad Software Inc). Simple linear regression was used to model the relationship between sound levels and ascending cut rates or vacuum pressures. Statistical significance was defined as *P* < .05 for all analyses.

## Results

The duration of vitrector use during surgical cases ranged from 5 to 15 minutes per case. The average sound level during vitrectomy ranged from 59.5 to 66.8 dBA. The maximum sound level recorded during vitrectomy was 88.2 dBA. Table 2 shows the data obtained for each vitrector system.

**Table 2.** Sound Pressure Levels During Vitrectomy Surgery by Vitrector.^a^

Parameter	Constellation Vitrector	Stellaris Vitrector	EVA Vitrector
Procedures	MH repair, MH repair, MH repair	VH, VH, ERM peel	RD, RD, ERM peel
Mean baseline OR sound level (dBA) ± SD	50.7 ± 0.4	53.1 ± 0.3	51.7 ± 0.4
Mean sound level (dBA) ± SD	60.5 ± 0.3	66.1 ± 0.8	59.4 ± 0.2
Mean maximum sound level (dBA) ± SD	76.5 ± 2.6	87.1 ± 1.3	68.6 ± 1.5
Mean minimum sound level (dBA) ± SD	51.7 ± 0.4	54.4 ± 0.5	52.4 ± 0.2
Duration of vitrectomy (min), range	7, 11	6, 15	5, 9
Time to exceed allowed daily noise exposure^b^ (h)	>8	>8	>8

Abbreviations: ERM, epiretinal membrane; MH, macular hole repair; OR, operating room; RD, retinal detachment; VH, vitreous hemorrhage.

a For each system, measurements were obtained from the average sound level produced during 3 vitrectomy procedures. All cases were performed using a 25-gauge cutter.

b As per Occupational Health and Safety guidelines based on case average sound level.

**Figure 2.**
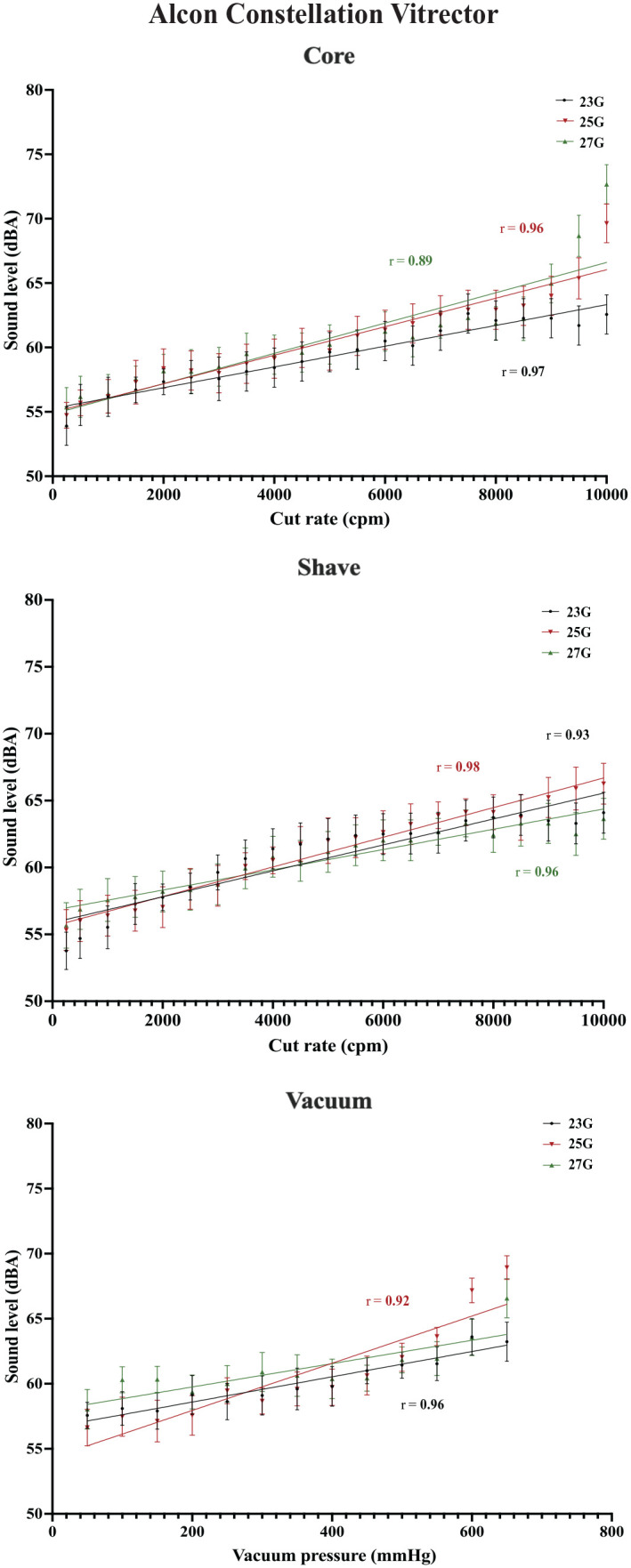
Cut rate and vacuum pressures for the Constellation vitrector with associated mean sound levels for the vitrector systems. The mean sound levels represent the average of 10 measurements at each cut rate or vacuum pressure. Correlation coefficients with associated lines-of-best-fit are plotted.

**Figure 3.**
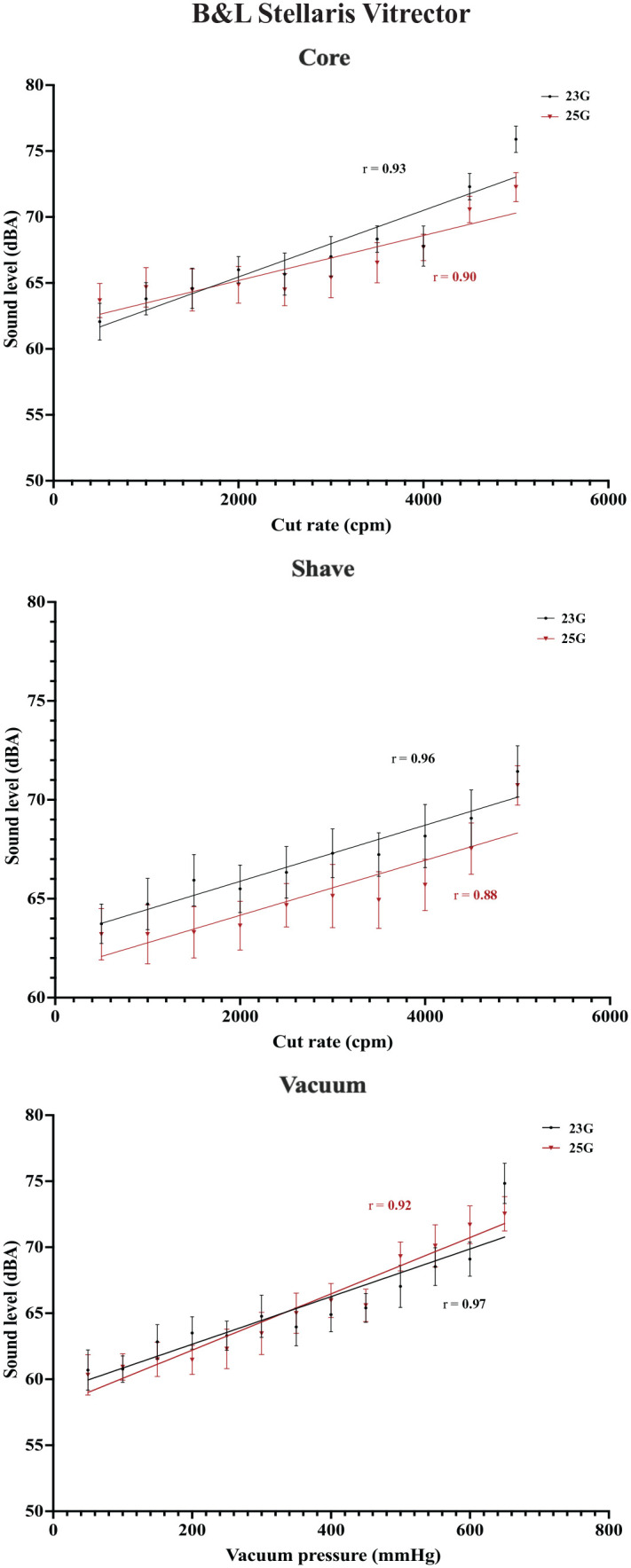
Cut rate and vacuum pressures for the Stellaris vitrector with associated mean sound levels for the vitrector systems. The mean sound levels represent the average of 10 measurements at each cut rate or vacuum pressure. Correlation coefficients with associated lines-of-best-fit are plotted.

**Figure 4.**
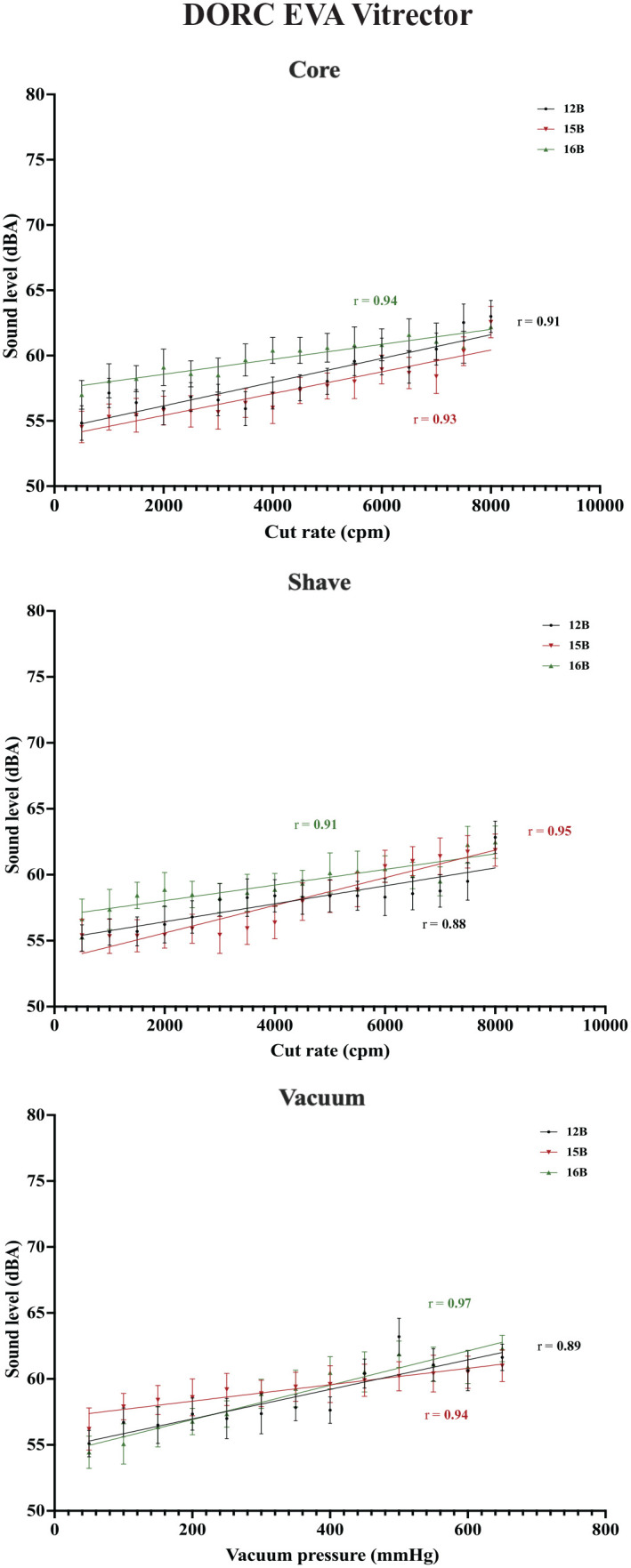
Cut rate and vacuum pressures for the EVA vitrector with associated mean sound levels for the vitrector systems. The mean sound levels represent the average of 10 measurements at each cut rate or vacuum pressure. Correlation coefficients with associated lines-of-best-fit are plotted.

An increasing cut rate was found to have a correlation with the sound level across the 3 vitrector systems (*P* < .001). All were strong positive linear correlations, with correlation coefficients (*r*) ranging from 0.88 to 0.98. With vacuum function in use, increasing vacuum pressures also had a strong positive linear correlation with sound level across systems (*r* = 0.83-0.97; *P* < .001). No difference was found between the various cutter gauges and associated sound levels (*P* > .05). Figures 2 to 4 show plots of the data for each vitrector system.

Of the sound levels produced by the vitrector machine that were measured, the maximum level recorded was 75.4 dBA during the use of VFC inject on the Constellation vitrector. Table 3 lists the settings and corresponding sound levels with the estimated time to exceed CCOHS and NIOSH guideline recommendations.

**Table 3.** Maximum Sound Pressure Levels Associated With Additional Settings by Vitrector Machine.

	Vitrector Sound Level (dBA)			Time to Exceed Allowed Daily Noise Exposure (h)^a^
Setting	Constellation	Stellaris	EVA	
Reflux	65.1	61.5	57.5	>8
VFC extract	72.3	—	63.5	>8
VFC inject	75.4	—	63.1	>8
Laser	70.1	62.2	58.5	>8
Diathermy	72.8	66.8	58.7	>8
Voice naming procedure	65.7	64.3	57.3	>8

Abbreviation: VFC, viscous fluid control.

a Based on US National Institute for Occupational Safety and Health and Canadian Centre for Occupational Health and Safety guidelines.

## Conclusions

This study evaluated the risk for hearing loss from vitrectomy by measuring sound levels in the OR from 3 of the most common commercially available vitrector machines. Although noise exposure has been evaluated in other surgical fields, to our knowledge no previous studies have assessed noise exposure in the ophthalmology OR.

Because of the limited duration of exposure, the noise produced in an OR does not typically pose a significant risk to patients. However, the potential impact of noise exposure is an important aspect of occupational health and should not be ignored for hospital staff. Canadian guidelines state that noise in excess of 87 dB requires mandatory hearing protective equipment in the workplace.^9^

In the present study, the mean sound level recorded during vitrectomy procedures ranged from 59.5 to 66.8 dBA, with a maximum sound level of 88.2 dBA. Sounds during the use of the vacuum function are produced by the vitrector tower, whereas the pneumatic guillotine cutter is responsible for sound emission during the use of the core and shave functions. Although the average sound levels during vitrectomy do not currently reach the limits of occupational health guidelines, our results suggest that increasing cut rates in future models could result in increased sound emission during the core, shave, and vacuum functions. This could pose a threat to hearing, in particular for surgical team members working in close proximity to the cutter or vitrector tower.

Not all harm from noise exposure is accounted for by sensorineural hearing loss. The World Health Organization (WHO) and the Environmental Protection Agency have suggested limits of 35 dBA and 45 dBA for hospitals and ORs, taking into account the impact of sound on surgical team activity interference.^10^ This includes considerations such as interference with effective communication and team performance.

In the field of otolaryngology, a systematic review of noise exposure in the OR found an overall mean noise level of 84.9 dBA. This level was deemed to be in excess of safety thresholds.^11^ Moreover, in a study examining noise levels in total knee and hip arthroplasty, the peak noise level during surgery measured more than 140 dB. Although noise exposure throughout these surgeries remained within occupational standards, the mean percentage of allowed daily noise reached 40% in the surgeons performing these cases. The authors ultimately recommended hearing protection during loud surgical steps or working toward quieter tools.^7^

Based on the findings of the present study, the sound levels currently produced during vitrectomy likely do not warrant immediate intervention. However, the association we found between the cut rate and the sound level suggests that increasing cut rates could produce noise that threatens hearing. Should sound levels reach hazardous levels, hearing protection may be warranted during vitrector use, as has been the case in other surgical specialties.

This study has several limitations. Given that each center operated using a single vitrector system, it was necessary to transport the dosimeter to the various sites to obtain data from multiple vitrectors. Device calibration was performed to mitigate changes that could have been induced by shipping. As expected, the baseline OR sound levels were similar but not identical, making comparison between vitrector systems difficult. The lack of data on the 27-gauge cutter for the Stellaris system is acknowledged. Unfortunately, 27-gauge packs were not readily available at the trial site at the time of the study.

In conclusion, given the emerging data on occupational noise safety in other surgical fields, this study sought to describe the risk for hearing loss during vitrectomy surgery. Current vitrector sound levels were not significant enough to cause noise-induced hearing loss. However, current sound levels may be sufficient for surgical team activity interference and therefore affect communication and performance as described by WHO recommendations. Furthermore, we found a strong positive correlation between the cut rate and the sound level, both from the vitrector tower during use of vacuum and from the pneumatic guillotine itself during the core and shave functions. This may threaten hearing should vitrector cut rates continue to increase and may be worthy of consideration in future development of surgical instruments.
